# Supplementary material for: Phytophthora sojae Effector PsAvh113 Targets Transcription Factors in Nicotiana benthamiana
Source: J Fungi (Basel). 2024 Apr 27;10(5):318. doi: 10.3390/jof10050318 (PMC11122517; doi:10.3390/jof10050318)
Supplement: Supplementary file 1 [file jof-10-00318-s001.zip › Supplementary Figures S1-S2.pdf]

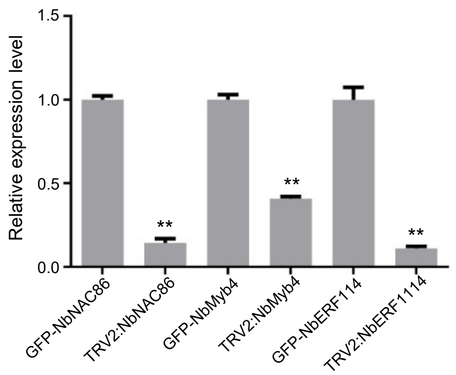

Figure S1. Relative transcript levels of *NbNAC86*, *NbMyb4*, and *NbERF114* genes in silenced leaves. RNA samples were isolated from leaves co-infiltrated TRV1 together with TRV2:GFP, TRV2: NbNAC86, TRV2: NbMyb4, and TRV2:NbERF114, respectively. *N. benthamiana Actin* gene was used as internal control. The experiment was performed in twice, with similar results. Asterisk indicates that there is a statistically significant difference **P<0.01, T-test).

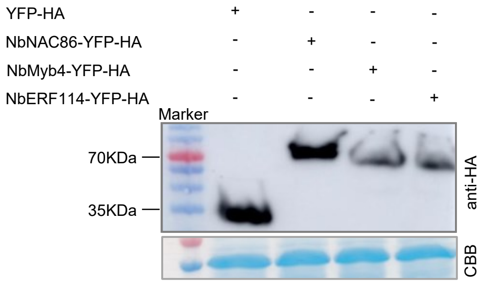

Figure S2. Immunodetection of three proteins in *N. benthanmiana* leaves expressing the *EV*, *NbNAC86*, *NbMyb4*, and *NbERF114* genes using anti-HA antibody, respectively. Coomassie brilliant blue (CBB) staining as a loading control for Western blot analysis.
